# Scaffolding the human partner by contrastive guidance in an explanatory human-robot dialogue

**DOI:** 10.3389/frobt.2023.1236184

**Published:** 2023-10-30

**Authors:** André Groß, Amit Singh, Ngoc Chi Banh, Birte Richter, Ingrid Scharlau, Katharina J. Rohlfing, Britta Wrede

**Affiliations:** ^1^ Medical Assistance Systems, Medical School OWL, Bielefeld University, Bielefeld, Germany; ^2^ Center for Cognitive Interaction Technology, CITEC, Bielefeld University, Bielefeld, Germany; ^3^ Psycholinguistics, Faculty of Arts and Humanities, Paderborn University, Paderborn, Germany; ^4^ Cognitive Psychology, Faculty of Arts and Humanities, Paderborn University, Paderborn, Germany

**Keywords:** HRI, XAI, negation, understanding, explaining, touch interaction, gesture

## Abstract

Explanation has been identified as an important capability for AI-based systems, but research on systematic strategies for achieving understanding in interaction with such systems is still sparse. Negation is a linguistic strategy that is often used in explanations. It creates a contrast space between the affirmed and the negated item that enriches explaining processes with additional contextual information. While negation in human speech has been shown to lead to higher processing costs and worse task performance in terms of recall or action execution when used in isolation, it can decrease processing costs when used in context. So far, it has not been considered as a guiding strategy for explanations in human-robot interaction. We conducted an empirical study to investigate the use of negation as a guiding strategy in explanatory human-robot dialogue, in which a virtual robot explains tasks and possible actions to a human explainee to solve them in terms of gestures on a touchscreen. Our results show that negation vs. affirmation 1) increases processing costs measured as reaction time and 2) increases several aspects of task performance. While there was no significant effect of negation on the number of initially correctly executed gestures, we found a significantly lower number of attempts—measured as breaks in the finger movement data before the correct gesture was carried out—when being instructed through a negation. We further found that the gestures significantly resembled the presented prototype gesture more following an instruction with a negation as opposed to an affirmation. Also, the participants rated the benefit of contrastive vs. affirmative explanations significantly higher. Repeating the instructions decreased the effects of negation, yielding similar processing costs and task performance measures for negation and affirmation after several iterations. We discuss our results with respect to possible effects of negation on linguistic processing of explanations and limitations of our study.

## 1 Introduction

Shaken, not stirred^1^—this is how the fictional British Secret Service agent James Bond prefers his martini cocktail. The catchphrase does not only indicate his preference, but also serves the purpose of contrasting it with the more common way of preparation. This form of contrastive explanation is a crucial element in ensuring effective communication and fostering understanding (Miller, 2019). This paper investigates how contrastive explanations from a robot affect the human in *Human-Robot Interaction* (HRI). Explanations within HRI serve diverse purposes, and one of their pivotal functions resides in facilitating task-based dialogues (Anjomshoae et al., 2019). Explanations primarily aim to improve transparency, cultivate trust, and boost confidence in systems that provide explanations [e.g., (Arrieta et al., 2020; Stange and Kopp, 2020; Matarese et al., 2021)]. In HRI, a major aim is to create a more natural explanatory dialogue between humans and robots to support humans in solving everyday tasks. In the present paper, we specifically investigate the use of contrastive explanations and ask whether negation has effects on the execution of instructed actions in human-robot dialogues.

### 1.1 Task-oriented dialogues with robots

#### 1.1.1 Guidance for task performance in HRI

A major field of robotic guidance for tasks comprises social robots for education, which are increasingly used as tutors or peer learners to improve foreign language learning, handwriting skills or chess playing (Belpaeme et al., 2018). One strand of research in this area addresses the question of how children can be motivated to spend effort and time on the learning task. The evaluation here is usually based on the assessment of whether such a social robot improves children’s task performance compared to a control group who learned without a robot (Van den Berghe et al., 2019). Saerbeck et al. (2010) have shown that social supportive behavior does not only yield higher motivation but indeed a higher learning success in learning an artificial language. However, Gordon et al. (2016) were not able to find such a facilitating effect of their motivation strategy on children’s learning success. Thus, it is unclear how motivation can support learning or understanding in detail. While motivation plays an important role in educational contexts, more specific guiding strategies may be more efficient in achieving understanding or task success. These strategies can guide the learner through attentional or temporal alignment strategies. For example, to address distraction during a task instruction, Carlmeyer et al. (2018) applied a hesitation strategy to regain the user’s attention. It could be shown that hesitations can indeed lead to higher task performance measured as post-interaction information recall (Richter, 2021). Guiding the learner through a task by temporal alignment has also been shown to be successful. Chromik et al. (2017) provided evidence that incremental (just in time) information presentation improves human task performance. Adaptation to the learner through an adaptive, gaze-contingent interaction strategy between robot speaker and human listener in a dictation scenario has also been shown to yield higher performance as compared to a non-adaptive rhythmic leading strategy (Palinko et al., 2015).

While these findings indicate that it is important to be adaptive to the (human) learner, the presented strategies all function on a surface, i.e., they take interactional features into account but do not integrate task knowledge. Clement et al. (2013) propose a more task-oriented guiding strategy by estimating the next learning step of school students in math exercises based on their performance on previous exercises. The system provides the students with exercises that it estimates can be accomplished by them because they are in accordance with their current competences or feasible learning effort. Evaluation showed that this approach yielded better learning performances by the students—in terms of correctly answered exercises of different levels of difficulty—than a tutoring system based on a non-adaptive strategy as specified by school teachers. In this approach, the guidance takes place by presenting complete exercises—or tasks—but without providing further detailed information about the task at hand or its relation to prior exercises. In general, there is little research on how to guide a learner within a new task or novel aspects of a familiar task or exercise to support her in her understanding of the task. In the following, we argue that negation is a strategy that can achieve a meaningful guidance.

#### 1.1.2 Negation in HRI

Linguistic negation is a grammatical construct that denies a supposition. Negations can be found in numerous everyday scenarios to contrast a positively affirmatively expressed proposition, including instances like instructions on how to open glass doors that are notoriously known to be moved the wrong way (“do not push but pull!”). The potential of task-related aspects of negation in the context of explaining (the robot uses it for contrasting) in HRI has not been explored so far. Studies often focused on narrow aspects of negation, such as affect or volition, as context conditions (Förster et al., 2019). Although recently the focus shifted towards explainable robots with some progress in the direction of explaining why robots reject commands of a human (Scheutz et al., 2022). In social robotics, negation is still generally seen as a device that imparts a “negative” attitude to the interaction and should therefore be avoided. In HRI research, the question of whether negation can enhance understanding has not been explicitly addressed—despite its potential—as we will demonstrate in the subsequent discussion.

### 1.2 Negation in human speech

#### 1.2.1 Negations

From the linguistic perspective on negation, the device of contrasting propositions is often used in explaining circumstances, be it personal preferences, causal chains or how things generally function (Miller, 2019). From an epistemic point of view, compared to a mere positive statement, a negation used in a contrastive utterance can narrow down the statement space, thereby specifying the question under discussion (Miller et al., 2017). Even if the question under discussion is semantically unambiguous, contrasting it with a hypothetical event might lessen the likelihood of confusion due to wrong presuppositions. Thus, on the one hand, a negation is enriching the proposition by providing previously excluded context which—although not desirable—constitutes a possible event. On the other hand, the added contrastive proposition is yet another proposition to process and even worse, it is a negated statement in its nature. In their overview article, Dudschig et al. (2021) examine linguistic negations and their influence on human performance.

#### 1.2.2 Processing cost

In the psycholinguistic literature, the phenomenon of negation attracted attention because it was found to cause higher processing costs [e.g., Kaup et al. (2007a; b)]. Processing of negation often leads to increased processing time (Tian and Breheny, 2019), even after extensive encounters of negation. Also, negation processing is more effortful and cognitively demanding (Deutsch et al., 2006). Processing costs have been explained by additional processing steps. Among the suggested mechanisms are tagging (negation requires mentally representing a core supposition and adding a negation tag (Clark and Chase, 1972)), inhibition of representations of responses (Beltrán et al., 2021), and conflict resolving according to which negations activate opposing representations and the conflict between them must be resolved (Dudschig and Kaup, 2018). Processing costs, however, vary depending on the context (Giora et al., 2007) and may even be absent, for instance in cases of short-time adaptation after processing a negated utterance or in certain pragmatic circumstances (Wason, 1965). Interestingly, negation as a linguistic phenomenon was found not only to cause higher processing costs but also to hamper recall [e.g., Mayo et al. (2004; 2014)], even plant false memories (Mayo et al., 2014), or elicit opposite actions (Wirth et al., 2019).

#### 1.2.3 Contextualization of negations: explanations

These results mainly stem from laboratory experiments in which negation was studied in limited contextual conditions, especially limited tasks such as responding to certain information. There is little empirical research on how negations are applied in natural settings, let alone on how they can be used in specific contexts such as explanations. This is surprising because in explanations the more competent partner attempts to provide important information to the addressee (the explainee). The fine-tuning of the information’s relevance to the explainee’s knowledge is often achieved by highlighting important parts (Axelsson et al., 2012) but also by limiting the explanation space. Negation is a highly successful means that limits the explanation space (e.g., Garfinkel, 1982; Köller, 2016).

Lining up with studies from psycholinguistics, we can propose that guidance with negative utterances has the potential to convey valuable information because—by negating a state of affairs—they address and relate to expectations (Kaup et al., 2007b), maintains attention on alternatives, and foster recall for the contrastive events (Singh and Rohlfing, 2023). These effects give reasons to assume that a negation obviously requires a person to reason beyond what is immediately present. One possible explanation for these effects is that negation is creating a contrast space, in which “possible worlds” (Garfinkel, 1982) get more in focus. The contextualization that is achieved by negation is an interesting effect that can be utilized for actions performed within an interactive task, making an explanation more successful. Our study investigates the application of negation in the specific context of explaining action execution for the purpose of task learning.

### 1.3 Tasks as context for explanations

#### 1.3.1 Tasks and actions

Above, we have argued for negation bearing the potential to convey valuable information when put into context. Within an interaction, a context can emerge from previous actions (Singh and Rohlfing, 2023), creating particular expectations about them. In fact, persons performing actions were found to construe a mental model of a sequence of actions that fits a task (e.g., Ballard and Hayhoe, 2009; Fusaroli et al., 2014). Lining up with previous research on actions, our findings on action understanding (Singh and Rohlfing, 2023) reveal that negation can be helpful because it addresses these expectations. Clearly, the context of action is multimodal. Trying to account for the complexity of this context, we reduced our action model in order to focus on manner and path.

#### 1.3.2 Manner and path

In topological terms, language has been shown to conceptualize an event in two main components: path and manner [e.g., Talmy (1975; 1985); Slobin (1987)]. The path component refers to the trajectory that the subject follows from its starting point to its destination, with reference to a ground or reference object. The manner component describes the specific way in which the subject moves along that path. The path component can either refer to the physical motion of an object along a trajectory, such as “The boy ran across the road,” or to a change in state, such as “The boy became happy to sad” (Jackendoff, 1985). In either case, the event involves a moving entity with a destination or goal. Studies in psycholinguistics have shown an asymmetry in how information about the source and goal of an event is encoded in memory (e.g., Lakusta and Landau, 2005; Papafragou, 2010). Specifically, the goal tends to be more salient than the source and the manner of the event, a phenomenon known as goal biases in event memory. A recent study of human-robot interaction found that the explainer only provides elaborated information about the manner of the motion when the explainee shows a sign of misunderstanding (Vollmer et al., 2013). This suggests that the way in which an event is perceived and approached is largely influenced by the conceptualization of its goal as opposed to manner, which is the primary driver of the interaction. Consequently, the importance placed on the manner in which the event unfolds may be comparatively diminished. According to Singh and Rohlfing (2023), employing linguistic negation as a means of providing contrastive guidance presents a potential method for mitigating goal biases. This approach may help to ensure that both the manner and the goal of an event are attended, improving the overall understanding of the event.

### 1.4 Research hypothesis

From our review of the existing literature, it becomes evident that a human–robot dialogue model can gain advantages by incorporating well-established explanation strategies from human–human communication. One effective approach to offer guidance within a context, particularly in a task where actions are required, is by making explicit references to the ongoing or emerging situation, including negating actions that were either possible or previously requested but should now be avoided. We need to stress that in the context of performing actions, explanations are similar to instructions because they address the way and manner of the performance rather than causal relations. Klein (2009) refers to this type of explanation as “how-explanation.”

Negations are known to address and relate expectations (Lüdtke and Kaup, 2006). They maintain attention on alternatives and foster recall (Singh and Rohlfing, 2023). The literature reveals that explicit negations in contrastive explanations (Miller, 2019), and when combined with actions, foster the human’s understanding in recall of these actions (Singh and Rohlfing, 2023).

Our work aims to address the gap in previous studies, which did not extensively investigate interactions guided by robots. We examined the impact of contrastive explanations on understanding and explore their potential to facilitate dialogue between a robot and a human. In pursuit of this objective, our focus is directed towards the facet of understanding that encompasses the capacity to execute an explanation while being scaffolded (Rohlfing et al., 2020). Therefore, we use contrastive guidance in a dialogue setting for an interaction study. Given the plethora of evidence on negation-induced processing cost and the rich contextual effect of negation at the same time, we put forth the following hypotheses:

**A)** We hypothesize that an utterance containing negation would require more processing time to react upon. This should be reflected in the overall reaction time to complete the task. Nevertheless, we acknowledge the rich contextual effect of negation when used to contrast the emerging expectations. Hence, we predict that in the contrastive instruction condition, participants will be quicker to adapt to the task in comparison to non-contrastive instructions and against the baseline. This adaptation should be reflected in both the similarity between the performed action by the human and the guided action by the robot as well as, the number of attempts needed to complete the task.

**B)** Verbal instructions featuring contrast will enhance comprehension, resulting in improved execution of instructed actions in comparison to non-contrastive and baseline conditions. To explore this, we will evaluate human performance through metrics, including the frequency of incorrectly performed initial actions, the number of attempts required to achieve correct gesture execution, and the similarity between the actions performed by humans as well as those guided by the robot (Section 2.5).

## 2 Materials and methods

This article presents an interaction study (in German language), designed as a (restricted) dialogue between a human and a robot, that investigated how negations can be used to generate contrastive explanations in the context of human-robot explanatory dialogues.

### 2.1 Participants

For this purpose a study with 31 participants (17 female, 14 male) was conducted. The age of the participants was in the range of 20–38 (*M*_*age*_ = 26.90). All participants were recruited on the campus of Bielefeld University (Germany) and from general mailing lists, containing also non-students. Participants’ average *Affinity for Technology Interaction* (ATI) (Franke et al., 2019) score was *M*_*ATI*_ = 4.00, SD_*ATI*_ = 1.07. An ATI of 3-4 refers to medium technology affinity.

### 2.2 Stimuli

#### 2.2.1 Tasks with different manner

**FIGURE 1:**
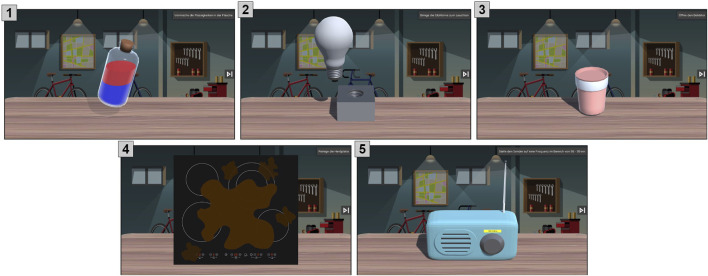
Objects (from 1 to 5: Bottle, Bulb, Cup, Hob, and Radio) for interaction on the touchscreen.

Floka (Lütkebohle et al., 2010), the virtual humanoid robot head, provided instructions (Table 1) for everyday tasks (Figure 1) that the participant had to carry out. These tasks involved common objects and were solved by executing a range of gestures on the touchscreen. A total of five distinct objects were designed for these tasks. The users’ task was to interact with these objects in two different manners (in the sense of actions). Each interaction manner was described by a verb and a corresponding touchscreen gesture. In each trial, Floka explained to the participant which specific gesture should be used for the task solution and described it verbally. During the interaction with an object in the correct way, the scenario provided participants feedback generated through alterations on the touchscreen, reflecting their interaction.

**TABLE 1:** Tasks with corresponding manners, required gestures and provided feedback by the scenario. Stimuli in German, English translations in brackets.

Description of objects and manner						
ID	Object	Manner I	Gesture I	Manner II	Gesture II	Feedback
1	Bottle	*schwenken* (*sway*)	twist	*schütteln* (*shake*)	slide	Fluids color changes
2	Bulb	*bewegen* (*move*)	slide	*anschubsen* (*push*)	swipe	Position changes
3	Cup	*schnipsen* (*flick*)	pinch	*drücken* (*press*)	hold	Lid opens
4	Hob	*scheuern* (*scrub*)	circle	*wischen* (*wipe*)	slide	Dirt disappears
5	Radio	*drehen* (*rotate*)	twist	*halten* (*hold*)	hold	Frequency changes

Each task, as visualized in Figure 1, was introduced by an overall verbal explanation of the goal of the task (e.g., “Mix the liquids in the bottle.,” “Open the cup.,” “Set the frequency of the radio in the range of 98–99.,” “Clean the hob.” or “Light up the bulb.”). For instance, the interaction with the object “bottle” could happen in two manners, *sway* and *shake*. The manner *sway* is represented by the gesture of a *twist* movement on the touchscreen. If the correct manner was used during the task, feedback for the participant was provided in the scenario in form of a color change by the fluids in the bottle. In the further scenarios, once the task was solved correctly, the bulb moved towards the pocket socket and the object lit up, the cup opened, and the hob became more visible as the dirt on the screen disappeared. For the radio, changing the frequency of the channel was visualized by a field changing on screen.

**FIGURE 2:**
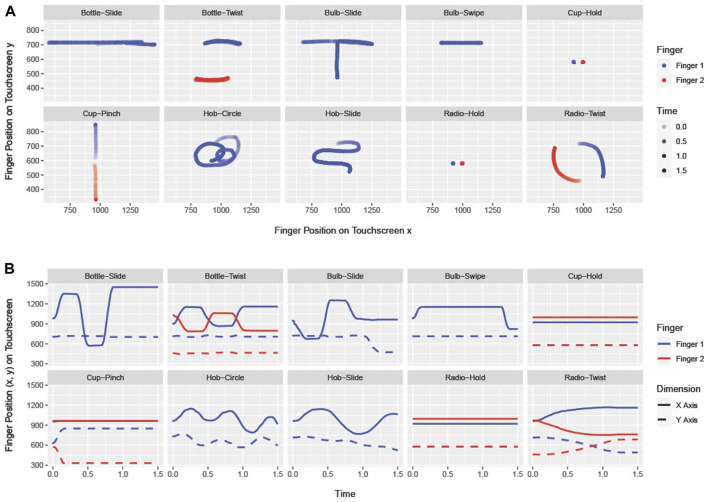
**(A)** Touchscreen gestures by finger inputs in 2D-space in which each axis represents one dimension. **(B)** Dimensions (x, y) by line type and touchscreen coordinate values over time on the y-axis.

Each manner was represented by a specific gesture, visualized as points on a 2D coordinate system (Figure 2). The twist gesture is a movement of two fingers sliding in a clockwise circular pattern, starting at opposite points. Sliding is represented on a touchscreen by the simple movement of one finger along a given path. Zooming in and out with two fingers in fast velocities describes the pinch gesture. For the circular gesture, participants had to move one finger in circular patterns on the touchscreen. A quick, jerky movement in a specific direction on the touchscreen, which is released at the target point, describes the swipe gesture. The hold-touch gesture is performed by two fingers that rest on two points on the touchscreen until a given threshold is exceeded.

#### 2.2.2 Verbal instructions

After the overall instruction concerning the task goal, Floka guided the participant by a contrastive or non-contrastive instruction for manner (Table 2). For a contrastive instruction, the robot contrasted the two possible manners for a task, the correct manner and a negation of the incorrect manner (“*now shake, not sway*” and *vice versa*). The non-contrastive condition included a placeholder (“*auf geht’s,*” “*let’s go*”) combined with the correct gesture (“*let’s go, now shake*” and *vice versa*). The verbal instruction by a single affirmation was the baseline instruction in the experiment. The baseline instruction was the only one presented in combination with an abstract object (see Figure 4). This generated three overall conditions for instructions and their corresponding instruction structures.

**TABLE 2:** Types of conditions and their verbal instruction structure with the original stimuli and translated examples.

Verbal instructions				
ID	Condition	Instruction structure	Stimuli (GER)	Example (ENG)
a	Contrastive	Affirmation-Negation	*jetzt [...], nicht [...]*	*now shake, not sway*
b	Contrastive	Negation-Affirmation	*nicht [...], jetzt [...]*	*not sway, now shake*
c	Non-Contrastive	Affirmation-None	*jetzt [...], auf geht’s*	*now shake, let’s go*
d	Non-Contrastive	None-Affirmation	*auf geht’s, jetzt [...]*	*let’s go, now shake*
e	Abstract Baseline	Affirmation	*jetzt [...]*	*now shake*

### 2.3 Experiment procedure

**FIGURE 3:**
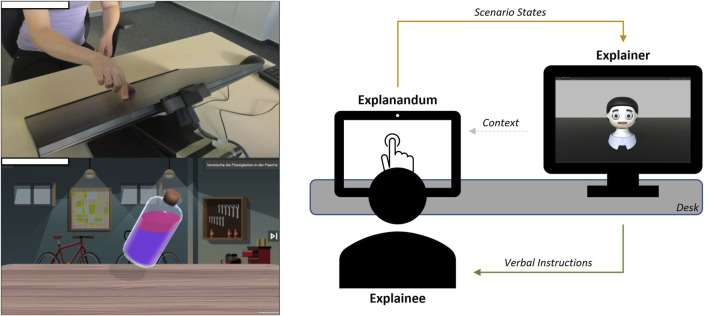
Floka (explainer) gives verbal instructions to the human (explainee) on how to solve tasks on a touchscreen (explanandum). Floka receives current state information about the task’s progress.

Figure 3 shows the experimental setup in which the stimuli (Section 2.2) in our HRI study were presented. Participants sat in front of a touchscreen. To their right, the virtual robot Floka was presented on an additional monitor. There was no human experimenter present. The experiment was presented automatically based on different states of the touchscreen application and was conducted in order to form a standalone dialogue between participant and Floka. For this purpose, an interface was developed that allowed the robot to react based to different states of the scenario (see Section 2.4).

**FIGURE 4:**
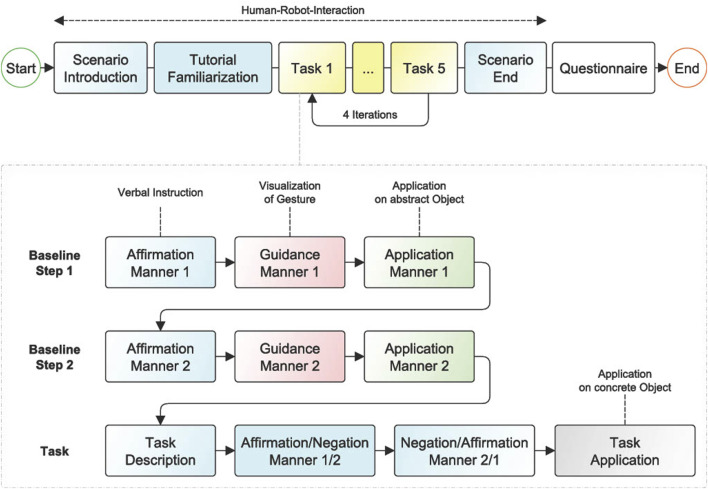
Experiment structure. Blue states are related to verbal instructions from Floka. The main experiment is separated into four iterations, with five tasks each.

All participants went through the experimental procedure (Figure 4) in a within-subject design. The selection of a within-subjects design aims to enhance statistical significance and reduce individual differences by accounting for potential confounding variables and experimental biases. This type of experimental design promotes these objectives by effectively randomizing verbal instructions and tasks, mitigating order-effects, and incorporating wash-out phases within the task gamification. First, the participants were instructed to read a prepared script, which contained general information about the course and structure, as well as the data protection declaration. The participants were positioned in front of the touchscreen and could initiate the experiment by clicking on the touchscreen. They conducted the experiment individually in a dedicated room where the robot and the scenario ran autonomously. The robot remained unresponsive to any questions posed by the participant. Once the study began, Floka provided a brief monologue explaining the structure of the study. The first task for each participant was to complete a small tutorial. This section included the familiarization with the touchscreen and allowed the participant to learn and practice the gestures which were required later. The aim was to create a realm of anticipations emerges, bridging the gap between verbal instructions and the forthcoming gestures to be executed. The tutorial comprised six tasks related to an abstract object (a cube), with one tutorial task assigned to each specific gesture. The participants had a maximum of 30 s per task to familiarize themselves with the gestures. Each task could also be ended prematurely before the time ran out. The tutorial was followed by the main part of the experiment.

The participant went through four iterations of a task series of five tasks each. Thus, there was a total of 20 tasks. The five different tasks in each iteration corresponded to the five conditions as described in Table 1. Each iteration contained every kind of task once, without presenting the last kind of task from the previous iteration as the first task in the following iteration. The robot supported the participant with verbal instructions and explained which gesture had to be used to solve the task. Two guidance-application steps, which served to create a link between gesture and verbal instruction (manner), were passed before each of the twenty tasks including the concrete object. Here, the robot demonstrated both possible manners with a simplified verbal instruction (“*now shake*”), followed by an application of the corresponding gesture by the participant on the abstract object (cube). Thereby, the participant was made aware of the two specific actions available for resolving the upcoming tasks. Visual guidance was implemented by the presence of one or two red dots on the touchscreen, simulating the finger movement necessary to solve the task. All demonstrated gestures maintained a consistent duration of 1.5 s. The participants had a maximum of 20 s to apply the demonstrated gestures to the abstract object. If the correct gesture had been used earlier, the next step started automatically. The guidance-application phase was used to further consolidate the mapping of verbal instructions and gestures during the study, visualizing the differences in the history of interactions between conditions.

After the two guidance-application steps, the main task began. First, Floka verbally explained the overall goal of the task to the participant. The verbal instruction strategies listed in Table 2 were employed to describe the gestures that should be utilized. Participants were given unrestricted time to solve the assigned tasks and had the option to terminate a task prematurely if they were unable to solve it. Verbal instructions by the robot were randomized within the tasks, providing an equal number of instructions for each condition in the experiment. Demographic information and subjective meanings about the experiment were asked in a questionnaire at the end of the experiment.

### 2.4 Technical setup

The HRI scenario (Section 2.3) with different tasks was developed with the Unity3D^2^ game engine. All object models were designed as 3D Objects in Blender^3^. The touchscreen event system in the application was implemented with the Lean Touch^4^ asset. This asset offers functionalities for manipulating objects or calculating events based on inputs on the scene using a touchscreen. A *State Chart eXtensible-Markup-Language* (SCXML)^5^ configuration was developed to run the experiment automatically in a state-machine like structure. This enables the execution of custom experiment orders and allows the reusability of the system. The configuration includes states, transitions, scenario-tasks, robot-tasks and functions for randomizing. SCXML-states correspond to different scenario-states of the experiment. State transitions describe the triggers that are used to change from one state to a subsequent state. Scenario-tasks and robot-tasks describe functions within a state. Each function triggers a behavior on the corresponding side (scenario or robot). For instance, a scenario-task could represent loading a new scene by given variables and a robot-task could execute a robot-specific behavior. Transitions are triggered by the respective callbacks of the functions, which allow the experiment to run automatically. A randomization function is used to exclude order effects within the experiment. For the HRI part of the experiment, we used a 3D simulation of the robot Floka. The simulation of the virtual robot was implemented in Unity3D, and it can interact with the participants on verbal and non-verbal communication channels. Floka can use verbal speech, express emotions through facial expressions and move its head and eyes. The *Robot-Operating-System* (ROS) (Quigley et al., 2009) is used to execute different behaviors on the robot and for exchanging information between the robot and the scenario. On the robot’s side, we used a modified architecture as middleware of an existing system to control different behaviors of the robot by given inputs from the scenario (Groß et al., 2022). These works allow the configuration of robot-behaviors in *eXtensible-Markup-Language* (XML) format and the execution of such behaviors via a network also for non-computer scientists (Schütze et al., 2022). The *Unity Robotics Hub* ^6^ was used to exchange information between scenario and robot via a *Transmission Control Protocol* (TCP)-connection via network. This allows the communication via ROS between the robot on the server-side and the touchscreen application as client. During the experiment, a logger captured data at a rate of 60 frames per second, including e.g., touchscreen event locations, scenario-states and robot instructions. Additionally, two webcams were utilized for video recording purposes: one for capturing the participant’s face and the other for recording hand gestures on the screen. Furthermore, screen capturing was employed to record the touchscreen along with the scenario. Finally, the software *SoSci Survey* ^7^ was used to collect personal data about participants and the experiment in a questionnaire.

### 2.5 Measurements

**FIGURE 5:**
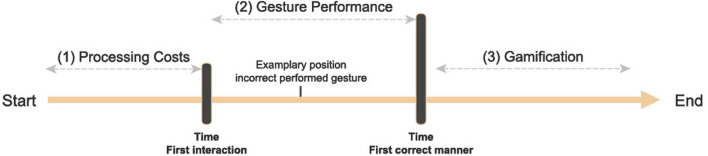
Timeline with points of measurements during a task. Timestamps for the first interaction with the touchscreen after a verbal instruction, and for the time until the first correct application of a manner.

The measurements in this experiment focused on two aspects. **(1)** The procedure for measuring the processing costs of the participants as reaction times in each task (Section 2.5.1). **(2)** The methodology for determining the manner-specific gesture performance (Section 2.5.2). Figure 5 shows the chronological sequence of the measurements for one trial of the experiment. A task was divided into three phases. 1) The time-frame from the beginning of the task (after receiving the overall instruction about the goal of the task) until the first interaction with the touchscreen by the participant constitutes the reaction time for this task. 2) The data recorded after the first interaction with the touchscreen until the execution of the first correct manner is the manner-specific gesture performance. 3) Any data that occurred after the point of the first correct manner is reported as part of gamification. This approach was intended to amuse the participants and provided a wash-out time before the next verbal instruction and task.

#### 2.5.1 Processing cost

This experiment deals with the influence of negation in explanations. One effect of negation is the additional processing loop and associated effects on participants’ reaction times (Section 1.2.2). After a verbal instruction pertaining to the overall goal of a task by the robot, each task consisted of an interaction with the touchscreen to solve the task. The time until the first interaction with the touchscreen was measured as the reaction time in each task (Figure 5).

#### 2.5.2 Manner-specific gesture performance

The present study focuses on evaluating the execution of a gesture in relation to a verbally instructed manner. For this experiment, precise data on the execution of individual gestures could be recorded in form of finger inputs, which allowed for the computation of correct vs. incorrect gestures and the measuring of insecurity regarding the execution of the gestures. The manner-specific performance of gestures was described by three characteristics. In a first step, the number of initially incorrectly performed gestures was considered in order to make a statement about how well an instruction could be understood. Secondly, the number of times the touch input was interrupted while performing a gesture was recorded. This number of attempts was used to draw conclusions about the continuity of a gesture. Thirdly, to evaluate the actual touchscreen inputs, a similarity between the demonstrated and performed gestures was calculated. For all these measurements, the period from the first interaction with the touchscreen to the first correctly recognized gesture was considered.

##### 2.5.2.1 Number of gesture and instruction matches

To identify the effect of an instruction on the execution of a gesture, the number of corrections was measured. This variable described the number of trials in which a participant initially made an incorrect gesture. An incorrect gesture is a gesture that does not follow the intention of the verbal instruction. For this, only the period from the first interaction with the touchscreen until the identification of the intention of a gesture was considered. A qualitative video analysis was carried out to evaluate the gestures first performed by the participant after hearing an instruction of the task. The video recording of the hand gestures and the screen were used for evaluation. Two independent annotators watched the video material and labeled the first gestures of each task for all participants. The annotation was performed blindly. The annotators had no information about the verbal instructions by the robot. They only knew which gestures were generally available to solve the task. For instance, for the task “hob,” the annotators knew that the robot could verbally introduce the gestures *scrub* or *wipe*. The annotators’ task was to describe the first recognized gesture out of the two possible gestures for each task. The comparison between the interpreted gesture by an annotator and the introduced gesture in the verbal guidance resulted in a match or mismatch.

##### 2.5.2.2 Number of gesture attempts

To measure the number of execution repetitions for the instructed gesture, the finger inputs on the touchscreen were regarded as a continuous data stream. Missing values in this data stream were interpreted as a lack of interaction with the screen. To assess the number of these breaks, the events in which the stream changes from interaction to no interaction were counted for the finger with the first contact on the touchscreen. These breaks were described as the number of attempts to perform a gesture.

##### 2.5.2.3 Gesture dissimilarity

The quality of a gesture performed by participants was determined by comparing it to the corresponding gestures presented by the robot. By considering the touchscreen inputs as two-dimensional points on the screen, the data points of a gesture could be described as a time series. *Dynamic-Time-Warping* (DTW) (Senin, 2008) allows for the comparison of two time series of different lengths by calculating the dissimilarity between them. For this purpose, DTW sets up a cost matrix between two data sets. Here, the distance (Euclidean distance) between the points of the first time series (query) and all other points of the second time series (template) is calculated. DTW calculates a local cost matrix for the alignment of two sequences x and y with the estimated distances (Eq. 1).

$$C_{l}\in R^{N\times M}:\;c_{i,j}=\|x_{i}-y_{j}\|,i\in \left[1:N\right],j\in \left[1:M\right]$$ (1)

$$c_{p}\left(X,Y\right)=\overset{L}{\underset{l=1}{\sum }}c\left(x_{nl},y_{ml}\right)$$ (2)

$$DTW\left(X,Y\right)=c_{p*}\left(X,Y\right)=\min\left\{c_{p}\left(X,Y\right),p\in P^{N\times M}\right\}$$ (3)

Once the local cost matrix is established, the calculation of warping paths takes place. The distances are summed up, and paths are computed based on the associated costs. One warping path with respect to the local cost matrix is described by Eq. 2. DTW refers to the outcome of the calculation involving the dissimilarity between the two time series. Therefore, the algorithm calculates the warping path with the accumulated minimum costs for all pairwise local costs (Eq. 3).

In general, DTW requires two defined time series of equal or different length. The demonstrated gestures, performed by the robot, have fixed lengths of 1.5 s. When the participants try to imitate these actions, there is no way of knowing where the start and end of the input of the actual gesture are. Each participant starts performing the gestures at different points in time and performs the movements with different speed and uncertainties. The performed gestures can be described as a continuous stream of data within the task. To allow a comparison between guidance and application, an approach is needed which can handle input streams with unknown lengths. In the literature, online DTW algorithms are used to calculate real-time measurements for continuous data streams (Sakurai et al., 2006). The problem of classifying gestures in real-time can be transferred to this problem. Parts of these concepts were used in a modified form to calculate the dissimilarity in the measurements of this study. Sliding windows are often used to perform an iterative process of comparing two time series (Li, 2015). To enable a comparison between the fixed time series (guidance) and the data stream (participant’s task), the entire task was considered in subsequences up to the execution of the first correct action. With the use of a sliding window, the time-series was divided into n subsequences by an iteration step of 0.1 s. Each subsequence, with the length of 1.5 s, was compared to the gesture to be imitated via DTW to calculate the dissimilarity. Therefore, n sliding windows with the length of 1.5 s were considered with a step-wise increase of 0.1 s on the entire time-frame until the first correct manner was recognized. Considering a frame rate of 60 s within the scenario and the logging, we aimed for an average number of 90 samples (touchscreen input events) in a sliding window.

In order to allow the use of DTW in this experiment, the following conditions have to be met: the time series of gesture coordinates has to be **(1)** made robust against interruptions in the data stream caused by the sensitive touchscreen, **(2)** made gesture comparison insensitive to amplitudes of different strengths and offsets and **(3)** shifted to the origin of the coordinate system by removing different starting positions. **(1)** In real data sets of this experiment, participants sometimes take breaks during gesture performance by pausing or lifting their finger for a short time. Changing the finger pressure on the touchscreen can also cause measurement interruptions. To ensure a continuous comparison using DTW despite interruptions, we removed the missing measurement points in the time series through interpolation (Lepot et al., 2017). Interpolation allows for a seamless comparison because the gestures have been associated with objects in the scenario and are not extended to different positions. **(2)** In addition to potential data point interruptions, we’ve also taken into account distinct prominent features within the time series when applying DTW. To achieve this, we made the time series less sensitive to varying amplitudes and offsets. Following a similar approach as in previous research (Rakthanmanon et al., 2012; Shokoohi-Yekta et al., 2017), we standardized the gestures in the guidance and every gesture within each subsequence of the performed gestures using z-normalization (Keogh and Kasetty, 2002). **(3)** The final assumption for comparing the guidance and the application involved eliminating the impact of varying starting positions of the gestures on the touchscreen. In our presented gesture data, the objects had starting positions that differed from those in the actual tasks for the participants. Differences in starting positions of the objects could have resulted in an offset when comparing gestures. This kind of offset—the distance from the coordinate system origin to the first touchscreen input of the gesture from both time series—was subtracted (Tang and Dannenberg, 2014).

## 3 Results

The analysis for this study focuses on the effects of contrastive, non-contrastive conditions against an abstract baseline (Table 2) and the corresponding subjective ratings provided by the participants during the experiment, as indicated in Section 1.4. In order to draw conclusions about participants’ performance during the experiment, results are presented across the iterations comprising the individual tasks or trials (Figure 4). All data processing steps and analysis for this study were performed in R (R Core Team, 2022).

### 3.1 Effects on processing cost

**FIGURE 6:**
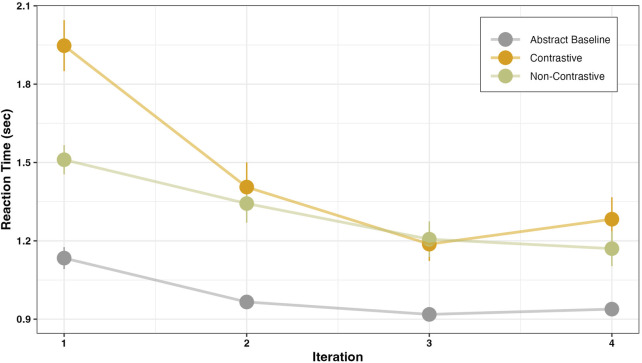
Visualizing the reaction time in seconds as a function of iteration count for each instruction condition.

We analyzed the data using a hierarchical mixed model approach in R (Bates et al., 2015), incorporating random slope adjustments for subjects and treating instruction type and iteration as fixed effect structures. We then added these structures gradually to a reduced model and tested the effect of each fixed variable using log-likelihood ratio tests following Barr et al. (2013). Based on these tests, we selected the best model. The results are visualized in Figure 6, and the model predictions are presented in Table 3.

**TABLE 3:** Reaction-Times: Parameter estimates of the fixed effects for instruction type and its interaction with iteration as the predictor.

Term	*β*	SE	*t*	*p*
(Intercept)	1.15	0.05	21.39	< .001
Iteration	−0.06	0.01	−4.93	< .001
Contrastive	0.87	0.08	11.10	< .001
Non-Contrastive	0.45	0.08	5.89	< .001
Iteration × Contrastive	−0.16	0.03	−5.72	< .001
Iteration × Non-Contrastive	−0.05	0.03	−1.80	.07
Contrastive × Non-Contrastive	0.42	0.09	4.30	< .001
Iteration × Contrastive × Non-Contrastive	−0.11	0.03	−3.14	< .001

Participants took significantly longer to perform the manner in both the contrastive (*β* = 0.87, SE = 0.08, *p* < .001) and non-contrastive (*β* = 0.45, SE = 0.08, *p* < .001) conditions when compared to the baseline condition. In a post-hoc pairwise comparison, in the contrastive condition the reaction time was significantly higher than in the non-contrastive condition (*β* = 0.42, SE = 0.09, *p* < .001). This delay may be attributed to the additional cognitive load introduced by negation processing in the contrastive condition. This result is in line with our predictions and previous studies stating that the negation processing might lead to a higher processing cost (Clark and Chase, 1972; Carpenter and Just, 1975; Kaup et al., 2007b).

Our main interest was to investigate the change in reaction time across iterations. In all instruction conditions, response times in the first trial were significantly higher than in subsequent iterations (*β* = −0.06, SE = 0.01, *p* < .001). Comparing the trial-dependent decrease in reaction time across each instruction condition, in the contrastive condition, participants initially took longer to respond in the first iteration. Their reaction time decreased significantly more rapidly in this condition, compared to both the baseline (*β* = −0.16, SE = 0.03, *p* < .001) and non-contrastive (*β* = −0.11, SE = 0.03, *p* < .001) conditions.

### 3.2 Effects on manner-specific gesture performance

Our previous results (Figure 6) have already shown that participants have to incur increased processing costs for contrastive instructions, which are reflected in longer reaction times during the beginning of a task. The following analysis focuses on participants’ execution of gestures based on instructions received from the robot. Three measurements are examined: **(1)** The initial correct intention of gesture execution, **(2)** number of gesture attempts and **(3)** the similarity between the executed gesture and the gesture demonstrated by the robot.

#### 3.2.1 Effects on number of gesture and instruction matches

The error rate in executing the first gesture can be used to describe an enhancement in gesture performance when correctly applying the gesture following a perceived instruction. A classification into a *match* or *mismatch* represents the result of the comparison between an annotator’s interpreted gesture and an introduced gesture in the verbal instruction by the robot. For this, two independent annotators qualitatively annotated all 620 tasks. The first annotation resulted in 550 matches and 70 mismatches (12.73%), while the second annotator evaluated it as 537 matches and 83 mismatches (15.46%). The agreement of the results of the annotations can be classified according to Landis and Koch (1977). Cohen’s *κ* was run to determine how large agreement between the two annotators was. The match was denoted as strong, *κ* = 0.86 (95% CI, .82 to .90) (McHugh, 2012). Upon receiving independent submissions from both annotators, the annotations were compared to identify discrepancies. A total of 44 cases with differing annotations were identified. Through a consensus process, the annotators reached an agreement on the final gesture under discussion, which results in 548 matches and 72 mismatches (13.14%). The annotated results were compared to the verbal instructions of the tasks to determine the overall number of matches.

**FIGURE 7:**
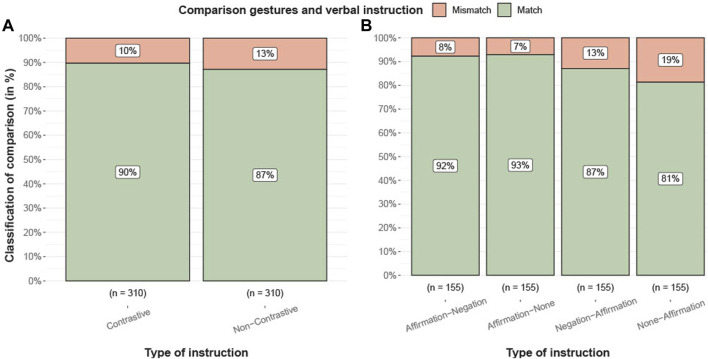
Comparison between first gesture by participants (agreement) and verbal instructed gesture from the robot. **(A)** Result for instructions in total and **(B)** more detailed structure of the different types. Visualizations using R-Package from Patil (2021).

Figure 7 describes the distributions for the instruction types in relation to the total frequencies of matches and mismatches. Based on the agreement of the annotators, the data is prepared for a chi-square test (Pearson, 1900). There was no significant association between the classification of matches of participants’ gestures and the robot’s instruction, *X* ^2^(1, *N* = 620) = 1.01, *p* = .32. The instruction types can be further subdivided based on their different structures to assess whether the lack of difference in the conditions can be attributed to verb placement effects. The total frequencies are then categorized into more detailed subgroups that correspond to these distinct instruction type structures (Table 2). There is a significant relationship between the classification of matches for gestures and verbal instruction, *X* ^2^(3, *N* = 620) = 13.20, *p* = .004. In a pairwise comparison, the instruction types *Affirmation-Negation and None-Affirmation* with *X* ^2^(1, *N* = 310) = 8.1231, *p* = .004 and *Affirmation-None and None-Affirmation* with *X* ^2^(1, *N* = 310) = 9.3, *p* = .002 show a significant difference.

#### 3.2.2 Effects on number of gesture attempts

**FIGURE 8:**
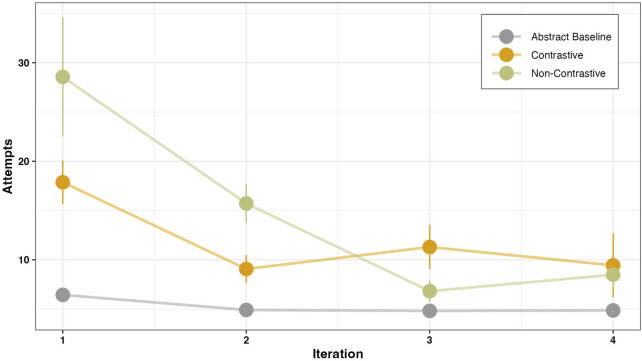
Plot showing the number of attempts before the first correct gesture for each instruction type and across trial iterations.

To assess the continuity of performed gestures, we analyzed the number of attempts before the first correct manner using a generalized linear mixed effects model, GLMER (Bates et al., 2015). The data was analyzed by treating the abstract condition as the baseline, and parameter estimates were obtained for each comparison by using post-hoc pairwise comparisons. The results are presented in Figure 8 and the estimates are provided in Table 4.

**TABLE 4:** Number of attempts: Fixed effects parameters for the number of attempts. The baseline (intercept) shows the values for the abstract baseline condition against which other conditions are compared.

Term	*β*	SE	*z*	*p*
(Intercept)	1.85	0.06	28.87	< .001
Iteration	−0.09	0.02	−5.86	< .001
Contrastive	1.11	0.06	19.63	< .001
Non-Contrastive	1.85	0.05	35.71	< .001
Iteration × Contrastive	−0.12	0.02	−5.55	< .001
Iteration × Non-Contrastive	−0.38	0.02	−17.58	< .001
Contrastive × Non-Contrastive	0.74	0.05	14.88	< .001
Iteration × Contrastive × Non-Contrastive	−0.25	0.02	−12.05	< .001

The instruction type has a significant effect on the number of attempts. Participants exhibited a higher number of attempts for both the contrastive (*β* = 1.11, SE = 0.06, *p* < .001) and non-contrastive (*β* = 1.85, SE = 0.05, *p* < .001) conditions when compared to the baseline. A post-hoc pairwise comparison conducted during the first iteration revealed that participants made significantly more attempts to perform the correct manner following the non-contrastive instruction when compared to the contrastive instruction (*β* = 0.74, SE = 0.05, *p* < .001). In terms of the iteration-dependent change in the number of attempts, we observed a general decrease in the number of attempts required to reach the correct manner across all conditions. When comparing the change in the number of attempts between the contrastive and non-contrastive conditions, we found that the non-contrastive condition led to a consistently higher number of attempts in the second iteration as well, which then decreased in the third iteration (*β* = −0.25, SE = 0.02, *p* < .001). The trial-dependent decrease in attempts suggests that the non-contrastive condition led to an overall faster decrease in the number of attempts required to perform the manner (*β* = −0.38, SE = 0.02, *p* < .001). This means that participants needed more trials to perform the first correct manner, but as trials progressed, they quickly adapted to the task. On the other hand, for the contrastive instruction condition, the overall attempts remained lower than for the non-contrastive condition.

#### 3.2.3 Effects on gesture dissimilarity

The dissimilarity was calculated based on the assumptions from Section 2.5.2 with the R-package for DTW by Tormene et al. (2008). A linear mixed effects model with varying intercept and slope by subjects was fitted to capture the iteration-dependent changes for the dissimilarity. Factor revealing was performed by treating the baseline condition as the intercept in the model, allowing for comparisons of all other instructions conditions (contrastive and non-contrastive) and their interaction with iteration. Additionally, a separate pairwise comparison was done between contrastive and non-contrastive conditions to get the estimated values of the main effects and its interaction with iteration. The model predictions and the estimates are shown in Table 5. The results show a main effect of instruction type such that the overall dissimilarity score was significantly lower in the baseline condition than in the contrastive (*β* = 39.34, SE = 5.22, *p* < .001) and the non-contrastive (*β* = 105.05, SE = 4.96, *p* < .001) conditions. Participants were more accurate in performing gestures on an abstract object. A pairwise comparison between contrastive and non-contrastive conditions at the first iteration revealed that the overall dissimilarity score was significantly lower in the contrastive condition than in the non-contrastive condition (*β* = −65.71, SE = 5.09, *p* < .001). This suggests that participants were more likely to be accurate in their gesture following a contrastive instruction in comparison to a non-contrastive instruction. An interaction of dissimilarity with the iteration suggests that participants’ dissimilarity decreases faster for contrastive (*β* = −11.58, SE = 2.04, *p* < .001) and non-contrastive (*β* = −30.30, SE = 1.93, *p* < .001) conditions when compared to the baseline condition. Importantly, the decrease in dissimilarity was faster in the contrastive than in the non-contrastive condition (*β* = 18.72, SE = 2.17, *p* < .001), which is also evident at the fourth iteration where the dissimilarity is minimum for the contrastive condition.

**TABLE 5:** Dissimilarity: Fixed effects parameters for the dissimilarity with instruction type and its interaction with iteration as predictor. The intercept shows the baseline condition.

Term	*β*	SE	*t*	*p*
(Intercept)	252.53	6.78	37.26	< .001
Iteration	−2.93	1.27	−2.31	.02
Contrastive	39.34	5.22	7.53	< .001
Non-Contrastive	105.05	4.96	21.17	< .001
Iteration × Contrastive	−11.58	2.04	−5.69	< .001
Iteration × Non-Contrastive	−30.30	1.93	−15.70	< .001
Contrastive × Non-Contrastive	−65.71	5.09	−12.90	< .001
Iteration × Contrastive × Non-Contrastive	18.72	2.17	8.62	< .001

### 3.3 Effects on subjective ratings

**FIGURE 9:**
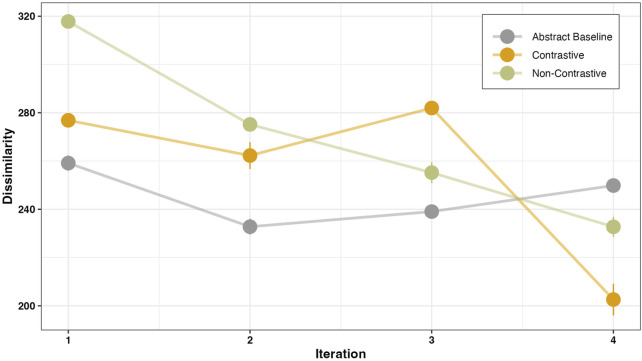
Mean dissimilarity before the first correct gesture. The plot shows the time-dependent changes to the dissimilarity values for different instruction types.

Previous results (Figures 8, 9) show that negation in explanations can also have positive effects on the execution of an instruction. Subjective perceptions of the instruction types by the robot were collected as part of the questionnaire. Participants rated each of the four instruction types on a seven-point Likert scale. For this purpose, a reformulated question similar to the Single Ease Question (Sauro and Dumas, 2009) was used.

**FIGURE 10:**
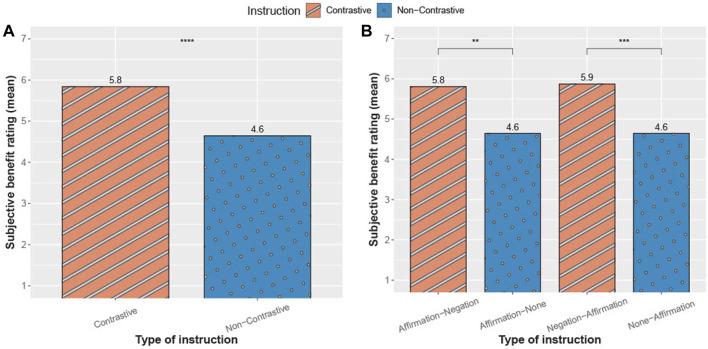
Subjective rating of the participant’s benefit from a verbal instruction. **(A)** with the comparison of instructions types overall **(B)** with their different structures. Rating from 1 (less helpful) to 7 (very helpful).

Figure 10 represents the mean values of the subjective evaluations across all participants in relation to the instruction types. A Wilcoxon test (Field et al., 2012) was conducted to evaluate whether contrastive instructions showed a greater subjective rating than non-contrastive instructions. The results indicate a significant difference, *p* < .01, with a moderate effect-size *r* = 0.379. In a second step, the instruction types were divided into their single structure types for considering the placement of the affirmation. Here, the aim was to assess the influence of verb placement on participants’ subjective ratings by examining its variation across the subgroups, while also identifying strengths and weaknesses in the instruction types’ structure. Therefore, another Wilcoxon test was conducted to evaluate whether the instruction types showed different subjective ratings. For the comparison of *Affirmation-Negation* and *Affirmation-None*, the results are significantly different, *p* < .01, with a moderate effect-size *r* = 0.402. In addition, for the comparison of *Negation-Affirmation* and *None-Affirmation*, the results show a significant difference, *p* < .001, with a moderate effect-size *r* = 0.353. These results emphasize that contrastive instructions subjectively helped participants to complete the tasks.

## 4 Discussion

Our study on a human–robot dialogue model was motivated by well-known explanation strategies from human communication. We proposed that one way to provide guidance in the context of actions is to relate to an ongoing or emerging context by explicitly negating actions that are possible or were previously requested but should now be avoided. We designed this guidance according to psycholinguistic research positing negation as a contrastive device, which relates to existing or emerging expectations and addresses these expectations (Kaup et al., 2007a). Our study provides a first attempt to investigate the role of negation as contrastive guidance in the context of HRI. In addition, our study extends current research on explanation strategies in human communication by putting negation into the context of joint actions.

To realize our scenario with joint actions, we employed real-world objects, each of which could be manipulated in two potential manners in order to accomplish the task goal in collaboration with the robot (Section 2.2.1). During these tasks, there is a potential predisposition towards a specific manner once the goal becomes clear, resulting in the default selection of a particular action among all the potential actions implied by the object. In such situations, an effective understanding model can be gauged by how efficiently one selects the correct manner while simultaneously keeping the task goal in focus. By incorporating a dialogue that introduces contrast through negation—explaining both the preferred approach for achieving the task goal and the alternative manners to be avoided—we created a more controlled selection process that is dependent on the desired task goal. In order to draw conclusions about the course of the interaction history and consider the effects during task repetition, the tasks were divided into iterations. This led to the execution of four iterations of task repetitions, where all instruction types were presented in combination with each task.

Validating this approach in relation to our hypothesis (Section 1.4), our analysis revealed that verbal contrast had two major effects on human actions: **(A)** While participants were overall slower in all the tasks that were accompanied by a contrastive explanation, their reaction time decreased at a faster rate with each iteration in this condition compared to the tasks with a non-contrastive explanation (Section 3.1). **(B)** The performance of an instructed action—as measured by number of attempts and gesture similarity—improved following a contrastive explanation when compared to a non-contrastive explanation (Section 3.2).

**A)** In the literature on negation, so far, processing costs were in the focus of investigation. When taking this measure into account and regarding the abstract baseline condition, both contrastive and non-contrastive instructions induced more processing costs on the participants in terms of reaction time (Figure 6). For the interpretation, we highlight that our investigation took place in the context of actions, in which a specific manner of action performance was considered as correct. Thus, the increase in processing cost is likely reflecting the perceptual complexity of the real objects (e.g., a bottle) in contrastive and non-contrastive instructions as opposed to the abstract object (e.g., a cube) in the baseline condition. In addition, an increase in reaction times following a contrastive instruction—providing information about both the correct and alternate manner—indicates that participants needed more time to process this additional information. Although the processing costs are high during the first iteration, participants demonstrated a quicker adaptation to the task demands in the contrastive condition. This supports previous findings, precisely that when negation is used in a context, the processing cost decreases (Tian and Breheny, 2019). Our results extend these insights to the context of actions. Overall, the observed adaptation following contrastive guidance appears to involve a contextual facilitation that leads to a rapid decrease in cognitive load.

**B)** Similarly, concerning the performance of an instructed action—as measured by number of attempts and gesture similarity—we observed that when contrastive guidance was provided the number of attempts required to reach the correct manner was lower in comparison to instances where non-contrastive instruction was given (Figure 8). A lower number of attempts indicates that participants needed fewer attempts to select the appropriate manner based on the task goal. One possible explanation for this phenomenon is that participants—when following the contrastive instruction—might have a greater sense of control in their selection of task-specific manners, and hence they relied less on a trial-and-error approach. These findings are in line with previous research indicating that negation engages neural mechanisms associated with higher-order action-monitoring processes (de Vega et al., 2016), as well as response inhibition and control (Beltrán et al., 2018; Dudschig and Kaup, 2018). Given that the two potential manners in our task were intrinsically linked to the task goal, it is more likely for participants to attempt both manners once the task goal becomes apparent, unless explicitly instructed not to perform one manner—as in the case of contrastive instruction. Previous research has demonstrated that when confronted with a negated instruction, individuals tend to mentally represent both the intended action and its alternate, leading to a two-stage processing strategy (Hasson and Glucksberg, 2006; Tian et al., 2016). This strategy, in our case, could potentially result in a higher number of attempts and a delay in the action selection process. However, according to the simulation account (Kaup et al., 2006; 2007b), the activation of the positive alternative in response to a negated instruction is short-lived—lasting only a few milliseconds—and diminishes rapidly after seconds. In addition, given the sufficient time for decision-making, the affirmative counterpart of the negated instruction may not necessarily translate into actual behavior and hence may not necessarily be acted upon as shown by Kaup et al. (2005, 2007a); Anderson et al. (2010); Scappini et al. (2015). Therefore, in our case it is plausible that while participants may have initially represented both the actual and alternate manners at the perceptual level—as indicated by the reaction time for the first action following the instruction—participants might have successfully suppressed the activation of the alternate manner at the decision level, where ample time was available for manner selection. As a result, they exclusively acted following the requested manner, requiring fewer attempts to converge towards it compared to the non-contrastive condition, where both manners were equally likely and required more attempts to arrive at the correct manner.

A lower number of attempts not only suggests a convergence between the executed and the instructed manner, but also indicates the requirement of fewer attempts performing the correct action, resulting in greater similarity at each iteration. This assumption was substantiated by the observed high gesture similarity following contrastive instructions as compared to non-contrastive instructions (Figure 9). Meaning, each action taken towards reaching the task goal was accompanied by a more precise manner following the contrastive instruction. The high gesture similarity following contrastive instructions further supports the notion that when participants were instructed to perform a manner that was contrasted with another (e.g., “now shake, not sway”), they initially took longer to process the instruction, as evidenced by the reaction time results. However, they required fewer attempts to accurately execute the correct gesture with enhanced precision. One possible explanation for this effect could be that participants were more mindful and deliberate in their manner selection when presented with contrastive instructions, as negation has previously been shown to recruit the domain general cognitive control processes (Beltrán et al., 2021). However, when provided with non-contrastive instructions, the participants relied more on a trial-and-error approach. The aforementioned assumption finds support in the evaluation of the participants’ subjective assessment. Participants rated contrastive instructions as being more helpful in solving the tasks (Figure 10). However, since our analysis did not directly examine the relational dynamics among reaction time, number of attempts, and similarity, we are cautious in speculating about the specific cognitive processes underlying manner initiation following each instruction.

Our study has also limitations regarding the range of manners that the objects could afford, which resulted in overall reduced complexity, as evidenced by the high success rates of 90% and 87% for the tasks following verbal instructions (Figure 7). Therefore, caution is needed when generalizing our findings to real-world scenarios. Further research will be needed to investigate if and under what conditions in more complex and natural contexts negation can even be more critical to the task success.

## 5 Conclusion

This study aims to address the existing research gap in the field of explanatory dialogues with robots, specifically focusing on the use of negations as a contrastive explanatory strategy within task-related contexts. The goal is to develop strategies that enhance the understanding of instructions and promote more natural dialogues with robots. We demonstrated that the positive effects of human interaction (Singh and Rohlfing, 2023) can be extended to HRI as well. Our research highlights that in both HRI and human interaction, the processing costs of negated instructions increase. However, even with sufficient contextual information for contrast, there is an advantage in terms of understanding the intended action. A primary objective in HRI is to enhance the naturalness of dialogues between humans and robots to foster human understanding and their actions. Explanation strategies, such as the use of negation, enable robots to effectively highlight specific aspects of an explanation capturing the interlocutor’s attention. This not only promotes more authentic interactions with robots but also paves the way for implementing adaptable robot models that can respond to individual circumstances based on the dialogue with humans. The utilization of negations empowers robots to steer conversations in a targeted manner, thereby improving their overall responsiveness.

These findings lay the groundwork for future investigations into interactive human–robot explanatory dialogues. A promising avenue for further research involves equipping robots with the ability to employ negation as an explanatory strategy, facilitating a deeper understanding of information in diverse situations. Furthermore, our results indicate that the impact of negation can be influenced by the complexity of tasks, highlighting the need for further exploration in this area. While this study primarily focused on negating the manner of an action, it is important to note that there are numerous other ways to shape explanations and instructions using negation. In our upcoming research, our objective extends beyond the micro-level of interaction, which focuses on individual linguistic items within an utterance (e.g., “not red”). Instead, our interest lies in the task-level, which pertains to the overall task itself (e.g., “do not pull”). This task-level perspective benefits from the cumulative history of interactions, encompassing established actions and tasks. This broader approach holds significant promise, particularly in the context of joint actions, and thus merits further exploration.

Our aim is to leverage our findings to introduce a methodology suitable for adoption by explanation-generation systems within the domain of *Explainable Artificial Intelligence* (XAI). This methodology highlights the potential not only to elucidate system algorithms, but also to effectively clarify actions within social contexts.

## Data Availability

The raw data supporting the conclusion of this article will be made available by the authors, without undue reservation.

## Footnotes

1 The catchphrase first appears in the novel Diamonds Are Forever (1956).

2 Unity3D https://unity.com [Accessed 21 March 2023].

3 Blender https://www.blender.org/ [Accessed 21 March 2023].

4 LeanTouch for Unity https://carloswilkes.com/#LeanTouchPlus [Accessed 21 March 2023].

5 State Chart XML (SCXML) https://www.w3.org/TR/scxml [Accessed 21 March 2023].

6 Unity Robotics Hub https://github.com/Unity-Technologies/Unity-Robotics-Hub [Accessed 21 March 2023].

7 SoSci Survey www.soscisurvey.com [Accessed 01 August 2022].
